# Effects of exercise on brain and peripheral inflammatory biomarkers induced by total sleep deprivation in rats

**DOI:** 10.1186/s12950-015-0102-3

**Published:** 2015-09-30

**Authors:** M. Chennaoui, D. Gomez-Merino, C. Drogou, H. Geoffroy, G. Dispersyn, C. Langrume, S. Ciret, T. Gallopin, F. Sauvet

**Affiliations:** Département Neurosciences et contraintes opérationnelles, Institut de recherche biomédicale des armées, Brétigny-sur-Orge, France; Université Paris Descartes, Sorbonne Paris Cité, EA7330 VIFASOM, Paris, France; Centre National pour la Recherche Scientifique, UMR 8249, 10 rue Vauquelin, 75005 Paris, France; Brain Plasticity Unit, ESPCI-ParisTech, 10 rue Vauquelin, 75005 Paris, France; Armed Forces Biomedical Research Institute (IRBA), B.P.73, 91223 Brétigny-sur-Orge, Cedex France

**Keywords:** Cytokines, Hormones, Brain, Periphery, Sleep deprivation, Exercise

## Abstract

**Background:**

Physical exercise induces neuroprotection through anti-inflammatory effects and total sleep deprivation is reported an inflammatory process. We examined whether 7 weeks of exercise training attenuates markers of inflammation during total sleep deprivation (24-h wakefulness) in the rat brain and periphery.

**Methods:**

Four groups of 10 rats were investigated: Sedentary control, Sedentary sleep-deprived, Exercised control, and Exercised sleep-deprived. Sleep deprivation and exercise training were induced using slowly rotating wheels and a motorized treadmill. We examined mRNA expression of pro-inflammatory (IL-1β, TNF-α, and IL-6) cytokine-related genes using real-time PCR, and protein levels in the hippocampus and frontal cortex, as well as circulating concentrations.

**Results:**

Compared to Sedentary control rats, hippocampal and cortical IL-1β mRNA expressions in Sedentary sleep-deprived rats were up-regulated (*p* < 0.05 and *p* < 0.01 respectively). At the protein level, hippocampal IL-1β and TNF-α and cortical IL-6 contents were higher in Sedentary sleep-deprived rats (*p* < 0.001, *p* < 0.05, *p* < 0.05, respectively). Peripherally, TNF-α, IL-6 and norepinephrine concentrations were higher in Sedentary sleep-deprived rats compared to Sedentary control (*p* < 0.01, *p* < 0.001, *p* < 0.01, respectively). Exercise training reduced the sleep deprivation-induced hippocampal IL-1β increases (mRNA expression and protein content) (*p* < 0.05 and *p* < 0.001), and TNF-α content (*p* < 0.001). At the periphery, exercise reduced sleep deprivation-induced increase of IL-6 concentration (*p* < 0.05) without effect on TNF-α and norepinephrine.

**Conclusions:**

We demonstrate that a 7-week exercise training program before acute total sleep deprivation prevents pro-inflammatory responses in the rat hippocampus, particularly the IL-1β cytokine at the gene expression level and protein content.

## Background

Mounting evidence suggests that sleep plays an important role in homeostatic restoration, thermoregulation, tissue repair, immune control, memory processing and brain plasticity [1, 2]. Conversely, controlled, experimental studies on the effects of acute sleep loss in healthy individuals have shown that mediators of inflammation are increased by sleep loss [3]. Sleep loss has been found to alter immune responses [4] and, in humans and rodents, to induce increases in circulating or brain levels of inflammatory markers such as interleukin (IL)-6, tumor necrosis factor-alpha (TNF-α) and IL-1β [5–7], and C-reactive protein (CRP) [8]. We previously suggested that, in rodents, the total sleep deprivation-related increase of TNF-α is responsible for endothelial dysfunction [9]. In humans, we have also demonstrated that one night of total sleep deprivation increases secretion of the proinflammatory cytokine TNF-α and is associated with significant sleepiness and diminished psychomotor performance [6]. In addition, Irwin et al. [10, 11] demonstrated that sleep loss induced monocyte production and transcription of IL-6 and TNF-α RNA messenger under the influence of the nuclear factor kappa-beta (NF)-κB transcription control pathway that play a key role in controlling cellular expression of pro-inflammatory genes. For this author, Toll-like receptors (TLRs) particularly TLR-4 play a role in the activation of NF-κB [12], and we showed increase in whole-blood TLR-4 mRNA in men during sleep loss [13].

The positive effects of exercise on many physiological systems, including the central nervous system (CNS), are well established [14, 15]. With rats, exercise training has been shown to improve memory decline related to aging or sleep deprivation and to decrease pro-inflammatory markers in the hippocampus [16–19], and also to decrease TNF-α and IL-1β levels in various brain regions of healthy young rodents [18, 20–22]. Four weeks of aerobic treadmill exercise attenuate the deleterious effects of sleep deprivation on long-term memory and hippocampal synaptic plasticity in adult rats [19, 23, 24]. In addition, Ma et al. [25] evidenced the protective effects of treadmill training against damage caused by cerebral ischemia through the down-regulation of cortical TLR-4 expression. In elderly men (e.g., large study of 4,252 subjects, 60 to 79 years old), Wannamethee et al. [26] observed that several hemostatic and inflammatory variables were dose-dependently and inversely associated with current physical activity.

The purposes of this study were (1) to characterize the effects of acute sleep deprivation (24 h of wakefulness) on mRNA gene expression and tissue content of pro-inflammatory cytokines interleukin-1 beta (IL-1β) and tumor necrosis factor alpha (TNF-α), known for their many physiological roles and their actions of sleep regulation [27], in the hippocampus and frontal cortex of rats, two regions of memory and motor performance [28], and also on the circulating concentrations; (2) to investigate the interest of a 7-week exercise training program to reduce central and peripheral inflammation. The TLR-4 mRNA gene expression in the brain was also assessed because, when down-regulated by long-term exercise, it has been suggested that this has a beneficial effect on chronic central and whole body inflammation [25, 29].

## Methods

### Animal care and experimental protocols

The experiments were performed using male Wistar rats (Centre d'élevage R Janvier, Le Genest-Saint-Isle, France) aged 4 weeks and weighing 125–130 g at the beginning of the experiments. The animals were housed five per cage under controlled conditions of temperature (20–23 °C), humidity (40 %) and light/dark cycle (12 h/12 h, lights on at 7 a.m) with ad libitum access to food and water. This experimental research has followed the french recognized ethical guidelines, and was approved under the number "Sauvet_10_1" by the animal committee of IRBA.The care and treatment of the animals was supervised by the veterinary surgeons of the Institut de Recherche Biomedicale des Armées (IRBA).

The animals arrived in the laboratory at least one week before the experiments. They were randomly allocated into one of four groups: a Sedentary control group (*n* = 11) allowed normal sleep; a Sedentary group (*n* = 8) with total sleep deprivation, an Exercise control group (*n* = 10) allowed normal sleep and an Exercised group (*n* = 8) with total sleep deprivation.

### Experimental training protocol

**Fig. 1:**
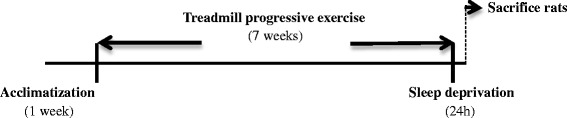
Schematic representation of the experimental protocol

The two groups of exercised rats were trained by running on a motorized treadmill for seven weeks. In order to minimize stress, the rats were progressively accustomed to the treadmill for one week (5 days, 10–15 min at a speed of 15 m/min). Animals reluctant to run during this training period were not used in the experiment. At the end of this period, the training program began: the rats were exercised 60 min each day, over a two-week period, at 18 m/min. The workload was then progressively increased until at the end of 5 weeks the rats were running at 25 m/min, 7 % grade, for 120 min per day and 5 days per week. This level of exercise remained stable during the final two weeks Fig. 1. During these 7 weeks, the rats were weighed three times a week. The rats belonging to the sedentary groups were placed daily in a new cage and also weighed three times a week [30].

### Experimental sleep deprivation protocol

The rats remained single-housed throughout the sleep deprivation (SD) experiments in activity wheels (Lafayette Instruments, IN, USA) as described and validated previously [31]. Briefly, these are large motorized stainless-steel activity wheels, with a 35.56 cm diameter, and internal wheel width of 10.9 cm. The rats were habituated to the activity wheel environment, including a 30 min period of activity wheel motion at 09:00 and 15:00 every day for 6 days before the experiments began. 24-hour SD (09:00 – 09:00) was achieved by the rotational movement of the activity wheel, programmed on a schedule of 3 s “on” at a speed of 3 m/min and 12 s “off”. Similar parameters have previously been shown to produce greater than 93 % wakefulness [9, 31].

In order to prevent acute exercise effects, the exercised rats were submitted to the sleep deprivation protocol after 24 h of recovery in their home cage.

### Sacrifice

The rats were weighed and then sacrificed by decapitation. Trunk blood was collected in vials and centrifuged at 3000 rpm for 10 min to separate plasma. The brain was immediately dissected on a cold plate. Brain tissue samples from the hippocampus and frontal cortex were placed in microtubes and either immersed in liquid nitrogen and stored at −80 °C for later cytokine content measurements or were submerged in RNAlater solution (LifeTechnologies, Carlsbad, CA, USA) for subsequent RNA isolation.

To check for normal growth patterns in the trained rats, we weighed their adrenal glands since adrenal hypertrophy in rats has been described as a potentially negative physiological adaptation to chronic, intensive exercise [30].

### Brain and blood concentrations of cytokines

Each tissue sample was thawed in 0.3–2.5 ml of ice cold buffer (pH 7.4, + 4 °C) containing 25 mM Hepes, 0.1 % CHAPS, 5 mM MgCl2, 2 mM AEBSF, 1 mM EDTA, 130 μM Bestatin, 14 μM E64, 1 mM Leupeptin, 0.3 μM Aprotinin and was then homogenized. The homogenates were centrifuged for 20 min at 16,000 g (+4 °C). Total protein content was measured in each supernatant using a Microplate BCA Protein Assay Kit (Pierce Biotechnologies, Rockford, IL, USA). Supernatants were aliquoted and stored at −80 °C until ELISA. Levels of TNF-α, IL-1β, IL-6 were determined using commercially available rat ELISA sensitive and ultra-sensitive kits (Invitrogen, Camarillo, CA, USA; R&D systems, Minneapolis, MN, USA). The assays were performed according to the manufacturer's instructions. All data are expressed as pg of cytokine per mg total protein to correct for differences in dissection size, except for plasma samples which are expressed as pg cytokine per ml plasma. The minimum detectable concentrations for TNF-α, IL-1β and IL-6 were respectively 0.7 pg/ml, 5 pg/ml and 16 pg/ml. The intra- and inter-assay CVs were respectively: 4.6 and 7.1 %, 3.9 and 5.7 %, and 6.5 and 14.0 %.

### mRNA isolation and reverse transcription

RNA isolation from brain tissue was performed using the Qiazol reagent method followed by RNeasy mini method on Qiacube (Qiagen, Venlo, Netherlands). A 50 μL final volume of RNA was eluted for each sample. A Nanodrop spectrophotometer (Nanodrop Technologies, Wilmington, USA) was used to quantify RNA in the extracts.

Reverse transcription was performed using the RT2 HT First Strand kit (Qiagen). The reaction was carried out from 1 μg of RNA. The cDNA was stored at −80 °C until use.

### Real time PCR

PCR was carried out with custom RT2 Profiler Arrays in combination with RT2 SYBR Green mastermix (SA Biosciences Qiagen, Venlo, Netherlands) using 1 μL of cDNA, in a 25 μL final volume. The cDNA sequences (rat) for IL-1β, TNF-α, and IL-6 were from GenBank (accession numbers NM_031512, NM 012675, and NM_012589 respectively), as were those of the two reference genes Hprt1 and Ppih (accession numbers NM_012583 and XM_345576). Reactions were performed on a LightCycler 480 (96-well block) and the crossing point values were calculated from LightCycler Software v3.5 (Roche Applied Science, Mannheim, Germany). Amplification specificity was checked using melting curve analysis following the manufacturer’s instructions. The genomic DNA control (GDC), the reverse-transcription control (RTC) and the positive PCR control (PPC) tested respectively for genomic DNA contamination, reverse transcription efficiency and the polymerase chain reaction. Assays for two housekeeping genes included in the arrays enabled normalization of data. The geometrical mean of housekeeping genes were calculated and used in normalized mRNA ± SEM levels.

### Blood hormone assays

Corticosterone, epinephrine and norepinephrine concentrations were determined in duplicate by enzyme-linked immunosorbent assays (ELISA) using commercial kits (IDS and LDN France). For corticosterone, the limit of sensitivity was 0.55 ng/ml, and the intra- and inter-assay CVs were respectively 3.8 and 7.7 %. Epinephrine and norepinephrine are extracted by using a cis-diol-specific affinity gel, acylated and then converted enzymatically. For these molecules, the assay limits of sensitivity were respectively 0.01 and 0.04 ng/ml, and the intra- and inter-assay CVs were respectively 5.0 and 13.0 %, and 8.5 and 16.1 %.

### Statistical analysis

In this study, we used one-way ANOVA followed by LSD post hoc test. Data are presented as means ± SEM. Spearman rank correlations were run between biochemical parameters in the hippocampus and frontal of all rats and respectively in the Sedentary and Exercised groups. All statistical analyses were conducted using Statistica 10.0, StatSoft Inc., Maisons-Alfort, France. For all statistics the significance level was set at *p* < 0.05.

## Results

### Body and adrenal gland weights

The body weight gain of Sedentary sleep-deprived rats was not statistically different compared to Sedentary control (270 ± 11 g vs 293 ± 5 g), while Exercised sleep-deprived rats was lower than Exercised control [244 ± 3 g vs 296 ± 11 g, F(3,33) = 8.17, *p* < 0.01].

The body weight gain of Exercised sleep-deprived rats was lower compared to Sedentary sleep-deprived (244 ± 3 g vs 270 ± 11 g, *p* < 0.05).

The weights of the adrenal glands were not statistically different in the four groups of rats (50.5 ± 4.7 and 51.0 ± 8.0 mg for Sedentary sleep-deprived and Sedentary control rats, 49.0 ± 3.0 and 50.5 ± 3.5 mg for Exercised sleep-deprived and Exercised control rats).

### Quantitative analysis of cytokines and Toll-like receptor (TLR)-4 gene expression in brain areas

#### Hippocampus

**Fig. 2:**
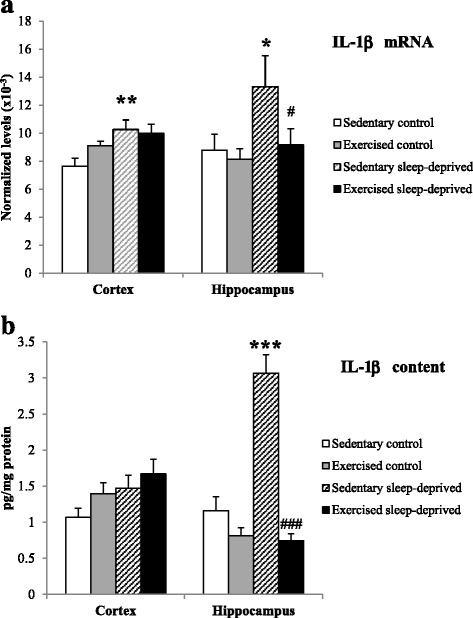
IL-1β normalized mRNA level (**a**) and protein content (**b**) in the frontal cortex and hippocampus of Sedentary control, Exercised control, Sedentary sleep-deprived and Exercised sleep-deprived rats. *Significantly different between Sedentary control rats and Sedentary sleep-deprived (*p < 0.05, **p < 0.01 and ***p < 0.001, respectively), # significantly different between Sedentary sleep-deprived rats and Exercised sleep-deprived (#*p* < 0.05 and ###*p* < 0.001), using one-way ANOVA analysis, *N* = 8–11 rats. Mean (±SEM) values are reported

**Fig. 3:**
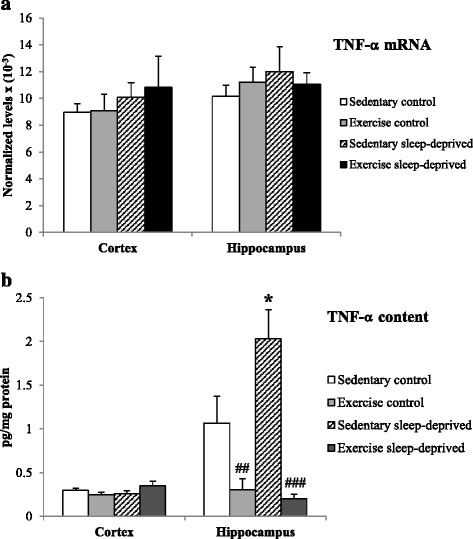
TNF-α normalized mRNA level (**a**) and protein content (**b**) in the frontal cortex and hippocampus of Sedentary control, Exercised control, Sedentary sleep-deprived and Exercised sleep-deprived rats. *Significantly different between Sedentary control rats and Sedentary sleep-deprived (*p < 0.05), # significantly different between Sedentary control rats and Exercised control (##*p* < 0.01) and between Sedentary sleep-deprived rats and Exercised sleep-deprived (###*p* < 0.001), using one-way ANOVA analysis, *N* = 8–11 rats. Mean (±SEM) values are reported

**Fig. 4:**
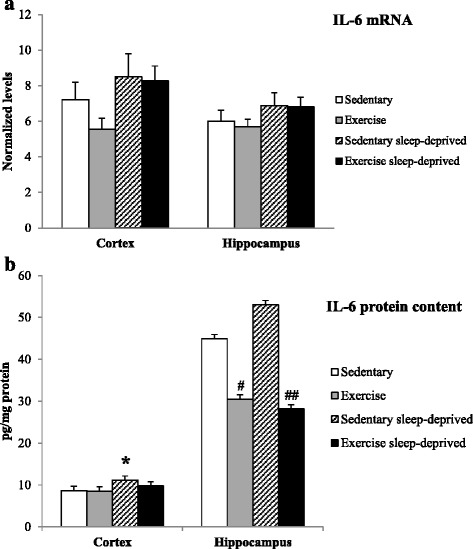
IL-6 normalized mRNA level (**a**) and protein content (**b**) in the frontal cortex and hippocampus of Sedentary control, Exercised control, Sedentary sleep-deprived and Exercised sleep-deprived rats. *Significantly different between Sedentary control and Sedentary sleep-deprived rats (*p < 0.05), # significantly between different Sedentary control rats and Exercised control (#*p* < 0.05) and between Sedentary sleep-deprived and Exercised sleep-deprived rats (##*p* < 0.01), using one-way ANOVA analysis, *N* = 8–11 rats. Mean (±SEM) values are reported

The IL-1β mRNA expression was higher in Sedentary sleep-deprived rats compared to Sedentary control rats [F(3,28) = 3.17, *p* < 0.05]. In addition IL-1β mRNA expression was reduced in Exercised sleep-deprived rats compared to Sedentary sleep-deprived rats (*p* < 0.05) Fig. 2a. No statistically significant changes in TNF-α and IL-6 mRNA expressions were observed between the four groups of rats (Figs. 3a and 4a).

TLR-4 mRNA levels were not statistically different [(31 ± 5)×10^−3^] and [(29 ± 5)×10^−3^] for Sedentary control and Sedentary sleep-deprived; [(25 ± 2)x10^−3^] and [(30 ± 3)×10^−3^] for Exercised control and Exercised sleep-deprived).

The correlation analysis showed that hippocampal IL-1β and TLR-4 mRNA levels were in significant and positive correlation within all rats and within the Exercised group (r = 0.422 and r = 0.733, respectively).

#### Frontal cortex

The IL-1β mRNA expression was higher in Sedentary sleep-deprived rats compared to sedentary control rats [F(3,28) = 4.16, *p* < 0.01] Fig. 2a. No statistically significant changes in TNF-α and IL-6 expression were observed between the four groups of rats (Figs. 3a and 4a).

TLR-4 mRNA levels were not statistically different [(27 ± 2)×10^−3^] and [(35 ± 2)×10^−3^] for Sedentary control and Sedentary sleep-deprived; [(28 ± 3)×10^−3^] and [(32 ± 3)×10^−3^] for Exercised control and Exercised sleep-deprived).

The correlation analysis showed that cortical IL-1β and TLR-4 mRNA levels were in significant and positive correlation within all rats and within Sedentary and Exercised groups rats (r = 0.858, r = 0.720 and r = 0.887, respectively).

### Cytokine contents (expressed in pg/mg protein) in brain areas

#### Hippocampus

The IL-1β and TNF-α concentrations were higher in the Sedentary sleep-deprived rats compared to the Sedentary control rats [F(3,33) = 34.4, *p* < 0.001 and F(3,33) = 11.6, *p* < 0.05, respectively]. IL-1β and TNF-α concentrations were lower in Exercised sleep-deprived rats compared to Sedentary sleep-deprived rats (*p* < 0.001, respectively). In addition TNF-α and IL-6 concentrations were lower in Exercised control rats compared to Sedentary control (*p* < 0.01 and *p* < 0.05) Figs. 2b, 3b and 4b. The IL-6 concentration was reduced in Exercised sleep-deprived rats compared to Sedentary sleep-deprived rats (*p* < 0.01) (Fig. 4b).

#### Frontal cortex

The IL-6 concentration was higher in Sedentary sleep-deprived rats compared to Sedentary control rats [F(3,33) = 3.31, *p* < 0.05] (Fig. 4b). No statistically significant changes in IL-1β and TNF-α concentrations were observed in the four groups of rats Figs. 2b and 3b.

### Plasma cytokine and hormone concentrations

TNF-α and IL-6 concentrations were higher in the Sedentary sleep-deprived rats compared to Sedentary control rats (F(3,33) = 5.75, *p* < 0.01 and F(3,33) = 5.12, *p* < 0.001, respectively). In addition IL-6 concentration was lower in Exercised sleep-deprived rats compared to Sedentary sleep-deprived rats (*p* < 0.05). The IL-1β concentration in the Sedentary control group was under the assay limit of sensitivity, and no statistically significant differences were observed between the groups of rats. Table 1.

**Table 1:** Cytokines (IL-1β, TNF-α and IL-6) and hormones (Corticosterone, epinephrine and norepinephrine) circulating concentrations in Sedentary control, Exercised control, Sedentary sleep-deprived and Exercised sleep-deprived rats

	IL-1β pg/ml	TNF-α pg/ml	IL-6 pg/ml	Corticosterone ng/ml	Epinephrine ng/ml	Norepinephrine ng/ml
Sedentary control	< 5.00	1.67 ± 0.29	18.7 ± 2.7	42.0 ± 19.3	5.28 ± 0.82	2.17 ± 0.23
Exercised control	8.51 ± 3.66	1.23 ± 0.40	28.6 ± 5.7	46.6 ± 12.8	4.09 ± 0.77	2.26 ± 0.21
Sedentary sleep-deprived	11.44 ± 7.02	3.85 ± 0.51**	41.9 ± 4.5***	79.2 ± 15.9	6.29 ± 0.86	4.03 ± 0.46^***^
Exercised sleep-deprived	16.71 ± 7.52	2.61 ± 0.74	27.6 ± 3.2***	71.2 ± 18.4	5.44 ± 1.31	3.48 ± 0.39^**^

"*" Significantly different between Sedentary control and Sedentary sleep-deprived rats and between Exercise control and Exercise sleep-deprived rats (**p < 0.01 and ***p < 0.001), "#" significantly different between Sedentary sleep-deprived and Exercised sleep-deprived rats (****p < 0.05), using a one-way ANOVA analysis, *N* = 8–11 rats. Mean (±SEM) values are reported

Plasma concentrations of norepinephrine were higher for Sedentary sleep-deprived rats compared to Sedentary control, and also for Exercised sleep-deprived rats compared to Exercised control (F(3,33) = 8.94, *p* < 0.001 and *p* < 0.01, respectively). No changes in concentrations were observed for corticosterone and epinephrine in the four groups of rats. Table 1.

## Discussion

In this study our objective was to investigate biomarkers of inflammation during a total sleep deprivation protocol (equivalent to 24 h wakefulness) in the rat brain and peripheral circulation, and the interest of exercise training as an anti-inflammatory therapy.

We quantified mRNA gene expression and tissue content of the main pro-inflammatory cytokines, TNF-α, IL-1β and IL-6, in two major brain regions involved in memory and motor performance, the hippocampus and the frontal cortex. The circulating concentrations of cytokines were also investigated as well as those of the stress hormones, corticosterone and catecholamines. Submitting rats to a total sleep deprivation protocol induced significant increases of IL-1β mRNA expression in the hippocampus and frontal cortex and increases in its protein level in the hippocampus. In addition, at the protein level, TNF-α in the hippocampus and IL-6 in the frontal cortex were higher after sleep deprivation. Peripherally, the pro-inflammatory response of sleep deprivation was seen mainly through increases in TNF-α and IL-6 concentrations, concomitantly to an increase in the concentration of norepinephrine. The second part of our study provides the first evidence that seven weeks of endurance exercise training reduces the increase of inflammatory brain markers induced by total sleep deprivation, mainly IL-1β mRNA expression and IL-1β and TNF-α protein concentrations in the brain hippocampus. At the periphery, exercise training reduces sleep-deprivation induced increase of IL-6 concentration.

The effects of acute sleep deprivation or sleep restriction on cytokine gene expression and protein levels in different regions of the rat brain have not been extensively studied. Mackiewicz et al. [32] and Taishi et al. [33] described increased levels of IL-1β mRNA in various regions of the rat brain (i.e., hypothalamus, hippocampus, cerebral cortex) with total sleep deprivation, while Wisor et al. [34] found no effects of short-term sleep deprivation (1 h and 3 h duration) on IL-1β, TNF-α and IL-6 mRNA levels in the brains of adult male mice. In another experiment, Wisor et al. [35] showed no effect of a 24-h sleep restriction on IL-1β, TNF-α and IL-6 gene expression in cells of the monocyte lineage into the mice brain. Recently, enhanced brain IL-1β and TNF-α mRNAs were observed after 1 day of 18 h of acute sleep loss, particularly in the frontal cortex [7]. In our study we show that the highest effect of sleep deprivation on the brain is to increase the gene expression of IL-1β, one of the two major cytokines involved in sleep-loss associated symptoms such as sleepiness and rebound sleep, fatigue, impaired cognition and memory [36]. This increase in IL-1β gene expression has been observed in the hippocampus, the major region involved in memory [28], and in the frontal cortex, a region of motor performance and where inflammatory cellular pathways are activated after stress exposure [37]. In this study we have confirmed that a change in IL-1 mRNA expression is not necessarily indicative of functional changes at the protein level as we found a significant increase in IL-1β protein levels with sleep deprivation only in the hippocampus. We suggested that the transcription, translation and the subsequent protein post-translational pathway could have been differently and regionally regulated under sleep loss. The significant increase of TNF-α protein in hippocampus and IL-6 in frontal cortex while their mRNA messengers were unchanged confirmed there is a cascade in cytokine production in the brain [38].

In rodents, using the slowly rotating wheel or the circular platform above water techniques for sleep deprivation it was found increased circulating concentrations of IL-1β, TNF-α and IL-6 [5, 39, 40], and also increased concentrations of corticosterone and norepinephrine (i.e., from 24 h to 96 h of sleep deprivation [41–43]. In our study, using the activity wheel protocol for sleep deprivation [31], we found that total sleep deprivation increased peripheral inflammation as the circulating concentrations of pro-inflammatory cytokines TNF-α and IL-6 were higher in sleep-deprived rats, concomitantly with norepinephrine concentrations. Nevertheless, levels of the stress hormones corticosterone and epinephrine were not statistically different between sedentary sleep-deprived and sedentary normal sleep rats; neither were the means for body and adrenal glands weights.

The present study is the first evidence reporting that exercise prevented total sleep deprivation-induced IL-1β increases at the gene expression and protein levels, and TNF-α increase at the protein level, in the hippocampus, a major brain region for memory. In the hippocampus, exercise training also decreases TNF-α and IL-6 protein contents in the control rats. The interest of endurance training exercise in preventing the brain inflammation induced by sleep deprivation has not been extensively studied. Vollert et al. [44] showed that a moderate treadmill exercise regimen for rats prevented anxiety-like behavior and brain oxidative stress (i.e., in the cortex, hippocampus and amygdala) normally induced by sleep deprivation. Recently, Zagaar et al. [19] demonstrated that a 4-week exercise training protocol may prevent hippocampal impairments of learning and memory induced by 24 h of sleep deprivation by preventing deleterious changes of BDNF and its associated signaling cascade.

The majority of studies have focused on the interest of exercise to limit age-related effects on pro- and anti-inflammatory cytokines and memory in the rat. Speisman et al. [18] evidenced that a 18-week protocol of conditioned wheel running improved memory and increased neurogenesis in aged rats but decreased hippocampal IL-1β and cortical VEGF protein concentrations, and that a negative correlation existed between IL-1β concentration and memory scores. In addition, and as we have demonstrated in our study in the sleep-deprivation condition, exercise clearly modulated neuroimmune factors in the cortex and hippocampus of aged rats which supports the notion that neuroimmune signaling in the brain is region-specific [16]. Lovatel et al. [17] showed that daily forced exercise (i.e., on a treadmill) for twenty minutes and over two weeks ameliorated aging-related memory decline and decreased pro-inflammatory markers, specifically TNF-α, IL-1β and NF-κB phosphorylated in the hippocampus, and that a negative correlation was found between IL-1β levels and performance in an aversive memory test. Chronic endurance exercise was suggested to represent a therapeutic tool to alleviate neuroinflammatory responses triggered by Tau abnormality in aged brains because it attenuates activation of microglia and astrocytes concurrently with the induction of TNF-α and IL-1β in mice which over expresses human tau23 in its brain [45]. Physical exercise has also been shown to provide endogenous neuroprotection as it reduces leukocytes infiltration and accumulation in the brain parenchyma in the setting of ischemia/reperfusion injury [46] and it reduces hippocampal TNF-α cell death signaling pathways in a chemical-induced injury model (i.e., using the prototypical neurotoxicant Trimethyltin, TMT) [20].

In this study we assessed TLR-4 mRNA levels because they were suggested to play a role during sleep loss in the activation of the NF-κB transcription pathway and the subsequent cellular expression of pro-inflammatory cytokines [11, 12], because we found increase of their whole-blood mRNA levels after total sleep deprivation in men [13], and also because treadmill training was demonstrated to reduce the overexpression of TLR-4 in rat brain tissue after ischemia and promoted functional recovery [25]. In the central nervous system, TLR-4 was preferentially found expressed in astrocytes and microglia under inflammatory conditions [47], and administration of a selective inhibitor of TLR-4 prevents increase of IL-1β mRNA in response to acute mild stress in the frontal cortex [48]. No significant effect of sleep deprivation was found on TLR-4 mRNA levels in the two brain regions, but significant and positive correlations existed with levels of IL-1β particularly in the exercised rats suggesting a possible regulatory role of TLR-4 expression. Further studies are in order to determine if activation of microglia and astrocytes and inflammatory cell infiltration in the brain is induced by total sleep deprivation concurrently with IL-1β and TNF-α, and the influence of exercise training.

Although our results are consistent with previous findings in rats, our experimental model of exercise training combined with sleep deprivation has limitation in its ability to generalize to humans. The two conditions are experimentally imposed using devices such as the slowly rotating wheel and the motorized treadmill as used in many previous studies, while human sleep deprivation or exercise training are usually voluntary or semi-voluntary. However, we have used the experimental model of total sleep deprivation of Christie et al. [31] that was demonstrated efficient to induce 24-h wakefulness without stress response (e.g., increase of corticosterone), and we have controlled the model for the non-specific effects of the activity wheel (i.e., locomotor activity and restraint) [9]. Despite this limitation, animal models of exercise training and sleep deprivation, including the current protocol, provide useful tools for investigating physiological mechanisms related to beneficial effects of exercise on the inflammation-related to sleep loss that cannot be studied readily in people.

## Conclusion

Our study has shown for the first time that seven weeks of physical training prevents the increase of pro-inflammatory cytokines induced by total sleep deprivation in the brain at the gene expression and/or protein level. Of particular interest was the reduction in the pro-inflammatory IL-1β cytokine mRNA expression and protein level, a cytokine involved in sleep-loss associated symptoms, in the hippocampus, the major brain region for memory. Further studies are warranted to determine interactions during sleep loss between increased inflammatory cytokines in the brain, memory impairments, and the interest of exercise training.
